# Beliefs about sleep paralysis in Turkey: *Karabasan* attack

**DOI:** 10.1177/1363461520909616

**Published:** 2020-03-29

**Authors:** Baland Jalal, H. Sevde Eskici, Ceren Acarturk, Devon E. Hinton

**Affiliations:** 1 University of Cambridge; 2 Şehir University; 3 Koç University; 4 Massachusetts General Hospital, Harvard Medical School

**Keywords:** cultural beliefs, sleep paralysis, Turkey

## Abstract

The present study examined explanations of sleep paralysis (SP) in Turkey. The participants were 59 college students recruited in İstanbul, Turkey, who had experienced SP at least once in their lifetime. Participants were administered the Sleep Paralysis Experiences and Phenomenology Questionnaire (SP-EPQ) in an interview. When asked whether they had heard of a name for SP, the vast majority (88%) mentioned the “*Karabasan*”—a spirit-like creature rooted in Turkish folk tradition. Seventeen percent of the participants believed that their SP might have been caused by this supernatural creature. Thirty-seven percent of participants applied various supernatural and religious methods to prevent future SP attacks such as *dua* (supplicating to God), reciting the Quran, and wearing a *musqa* (a type of talisman inscribed with Quranic verses). Case studies are presented to illustrate these findings. The *Karabasan* constitutes a culturally specific, supernatural interpretation of the phenomenology of SP in Turkey.

## Introduction

Sleep paralysis (SP) is characterized by involuntary atonia occurring either when one is about to fall asleep or when waking up (i.e., constituting a brief period of complete skeletal muscle paralysis accompanied by semi-consciousness; e.g., American Academy of Sleep Medicine, 2005; Hobson, 1995; Kandel et al., 2000; Paradis et al., 2009). During Rapid Eye Movement (REM) sleep, we might experience vivid dreams, events that are “remarkably faithful replica[s] of waking life” (Snyder, 1970, p. 133). If we were to act out these dreams, we would not only interrupt our sleep but also run the risk of hurting ourselves, as observed in REM sleep behavior disorder (Frauscher et al., 2007; Wing et al., 2008; Zhang et al., 2008). The human brain has an ingenious solution: The dorsolateral pons and ventromedial medulla of the brain stem comprise a system that suppresses skeletal muscle tone during REM sleep, leaving the entire body temporarily paralyzed (for details, see Brooks & Peever, 2012). The perceptual and motor aspects of REM sleep, however, can sometimes decouple, and the person will begin to wake up without being able to move or speak as the muscle paralysis or atonia has not yet waned (Paradis et al., 2009). SP can occur as a symptom of narcolepsy, a rare autoimmune sleep disorder (Levin, 1933; Ohayon et al., 2002). But the vast majority of SP episodes are unrelated to sleep pathology (Fukuda et al., 1987; see also, Hufford, 1982; Sharpless & Doghramji, 2015). Lifetime rates of SP across published studies (i.e., often conducted in Western countries) have been estimated around 20% (Sharpless & Barber, 2011). SP rates differ across ethnic, cultural, and racial groups (Jalal & Hinton, 2013, 2016; for a review, see Sharpless & Barber, 2011). Moreover, elevated rates of SP have been linked with psychopathologies such as post-traumatic stress disorder (PTSD) (Hinton et al., 2005b; Ohayon & Shapiro, 2000; Yeung et al., 2005), panic disorder (Bell et al., 1986, 1988; Friedman & Paradis, 2002; Paradis & Friedman, 2005; Yeung et al., 2005), generalized anxiety disorder and social anxiety (Otto et al., 2006; Simard & Nielsen, 2005), and also anxiety sensitivity (Ramsawh et al., 2008).

The vivid and often terrifying dreams of REM sleep can occasionally spill over into emerging wakefulness, which is like having a nightmare with one’s eyes wide open. These hallucinations during SP, either hypnagogic (during sleep onset) or hypnopompic (sleep offset), are of multiple types: seeing approaching human-like shapes, hearing footsteps, experiencing levitation and autoscopy, i.e., out-of-body experiences. These hallucinations often create the perceptual experience of a menacing intruder, which may be seen, sensed, or heard. In particular, SP experiencers tend to hallucinate a human-like shadowy figure approaching the sleeper, which sits on the sleeper’s chest and suffocates them (Cheyne & Girard, 2009; Cheyne et al., 1999a, 1999b; Jalal & Ramachandran, 2014, 2017; Nielsen, 2007; Simard & Nielsen, 2005; Solomonova et al., 2008). These general characteristics of the experience are reported worldwide, regardless of cultural context (Hufford, 1982, 1995, 2005), and can therefore be understood as being driven by the underlying neurobiology. Unsurprisingly, SP often causes great fear and terror (Cheyne & Pennycook, 2013; Jalal & Hinton, 2013; Schredl & Doll, 1998; Sharpless et al., 2010; Solomonova et al., 2008).

Each culture provides its own interpretations of the experience of SP, and REM mentation may help give elaborate hallucinations culturally distinctive features. Supernatural interpretations of SP are the most common, even in urbanized, secular societies (Bloom & Gelardin, 1985; Hufford, 2005; Jalal et al., 2015). Sometimes, even after learning about the neurobiological basis of SP, experiencers still adhere to supernatural explanations (Jalal et al., 2014a). Transculturally, examples of supernatural accounts of SP include “Old Hag” in Newfoundland (Hufford, 1982; Ness, 1978); “*Kanashibari*” in Japan (i.e., demons; Arikawa et al., 1999); “ghost oppression” in China (Wing et al., 1994); “the ghost pushes you down” among Cambodians (Hinton et al., 2005a, 2005b); space alien abduction among one subgroup in the United States (McNally & Clancy, 2005); the “*Pandafeche* attack” in Italy (Jalal et al., 2015); and the “*Segatelelo* assault” (caused by black magic and demonic dwarf-like creatures called the “*Tokoloshe*”) among some South African cultural groups (Jalal et al., 2018).

A study conducted on the phenomenology of SP in Egypt, a Muslim majority country, found that the vast majority of experiencers (71%) interpreted their SP as a supernatural event. Nearly half (48%) of these experiencers believed their SP to be caused by the *Jinn* (Jalal et al., 2014a), which are spirit-like creatures in the Islamic tradition that have the ability to possess, harm, and even kill their victims (Jalal et al., 2014a; on the *Jinn*, see also Amer & Jalal, 2011). The authors also found that many SP experiencers in Egypt (41%) resorted to “Islamic medicine” (*ruqyah*, a form of traditional healing) to prevent future attacks by the *Jinn* (i.e., to prevent SP), including reciting the Quran (e.g., over water and then pouring that water on the body for protection) and engaging in ritualistic prayer. In addition, it is not uncommon for SP experiencers in Egypt to consult a local Muslim priest (i.e., an *imam* or *sheikh*) for advice about their experience (Jalal et al., 2014a).

Cultural interpretations and beliefs about SP are crucial, as they may shape aspects of the experience. According to the “salience hypothesis,” SP takes on greater salience when interpreted through a specific cultural filter (Spanos et al., 1995). For instance, higher rates of SP are usually reported in cultural groups where people share information about the experience; that is, discuss its causes and remedies. Indeed, the availability of such cognitive categories could affect the level of attention paid to otherwise ambiguous events, such as REM paralysis sensations (Neisser, 1976). This phenomenon has also been referred to as “cultural priming.” In other words, individuals in these cultures are primed to readily recognize subtle and ambiguous paralysis cues and then seek to confirm these by attempting to move (Spanos et al, 1995). “Cultural priming” may entail culture-driven fear of the experience, as an elaborate supernatural attack. This would further motivate individuals to try to escape the event. However, attempting to move during SP might be problematic. The panic-hallucination (PH) model of SP (Jalal, 2016) hypothesizes that great fear of the experience (e.g., owing to cultural beliefs about SP) will lead to a panic-like reaction, resulting in the sleeper attempting to move to overcome the paralysis. Attempting to move in the absence of dampening proprioceptive afference (in effect, telling the brain to adjust the level of muscle clenching) could exacerbate unpleasant somatic sensations, such as bodily tightness and chest pressure, and trigger pain and spasms in limbs (see also Cheyne et al., 1999b; Jalal & Ramachandran, 2014). This could then feed into the content of hallucinations and possibly prolong the immobility. It is even thought that “terrorized immobility” could represent a trauma cue generating conditioned fear of SP—resulting in amygdala hyper-activation, and thereby triggering a positive feedback loop (Bell et al., 1988; Hinton et al., 2005b; Ohayon & Shapiro, 2000; Paradis et al., 1997). Cheyne and Girard (2009) have proposed a related model that likewise emphasizes the role of atonia-induced fear and threat-hypervigilance systems in regard to influencing hallucinations. Nielsen (2007) provides another model stressing social imagery vis-à-vis sleep paralysis hallucinations. Increased arousal from catastrophic cognitions and/or trauma recall during SP events may increase night-time awakening during REM sleep, leading to more night-time awakening, and in turn predisposing the sufferer to more SP (Hinton et al., 2005a, 2005b; Jalal & Hinton, 2013); particularly if these terrifying episodes lead to or worsen pre-existing chronic anxiety, causing further sleep disturbances.

The idea that SP could serve as a trauma cue dovetails with McNally and colleagues’ findings (McNally et al., 2004). The authors reported that individuals who claim they were abducted by space aliens (but ostensibly had undergone SP) showed elevated psychophysiological reactivity to audiotaped scripts describing their “alien encounters”; this reactivity was either comparable to, or even exceeded, the physiological reactions of PTSD sufferers listening to audiotaped descriptions of their traumatic experiences. Such “cultural priming” also seems to occur among Cambodians, who share elaborate supernatural explanations of SP as a dangerous ghost visitation; members of this group have very high rates of SP and reported long durations of immobility during the event (5.3 minutes) (Hinton et al., 2005b). Indeed, it was found that 49% of Cambodians had experienced SP in the last year, and that almost all of these experiencers (i.e., 45/49 or 92%) had experienced four or more episodes in the last year. Another noteworthy example is Egypt, where, as mentioned, many experiencers in the general population understand and discuss SP in the context of *Jinn* attacks and often seek out traditional healing remedies to get rid of the attack (Jalal et al., 2014a). Like Cambodians, Egyptians report very high lifetime rates of SP (i.e., an average of 19.4 episodes in a lifetime) and long durations of immobility (5.2 minutes) (Jalal & Hinton, 2013). Moreover, among Egyptians, SP is associated with extreme terror and fear of impending death in 50% of experiencers (Jalal & Hinton, 2013). By comparison, in Denmark—a country where there are no such elaborate cultural traditions (i.e., the event is regarded as an odd physiological event; Jalal et al., 2014a)—lower lifetime rates of SP have been reported (experiencers experience an average of six SP episodes in their lifetime). Danish experiencers also report shorter immobility during episodes (4.2 minutes) compared to Egyptians (Jalal & Hinton, 2013). In Denmark, only 17% of experiencers fear dying from SP, which is less than among Egyptians. Consistent with the salience hypothesis and unlike in Denmark, in Egypt, believing SP to be caused by supernatural forces is significantly associated with fear of the experience and with longer duration of immobility (on cultural salience, see also Hinton, Hufford et al., 2005). In short, among Egyptian experiencers in the general population, unlike those in Denmark, SP may take on greater salience, potentially leading to increased rates, longer periods of immobility, and excessive fear that could in some cases possibly trigger psychopathology symptomatology, such as chronic anxiety.

To our knowledge, no studies to date have explored cultural explanations and beliefs about SP in Turkey—which, like Egypt, is a Muslim majority country steeped in Islamic beliefs and tradition. In the current study, we explore cultural interpretations and causal explanations of SP and related practices and traditions in Turkey. We do so using the Sleep Paralysis Experiences and Phenomenology Questionnaire (SP-EPQ; Jalal et al., 2015), and present some cases.

## Methods

### Participants

The participants were Turkish undergraduate college students (*N = *59) recruited from İstanbul Şehir University, a private university located in İstanbul, Turkey. Research participants had experienced SP at least once in their lifetime. Seventy-five percent (44/59) of participants were female; and the age of participants ranged from 20 to 37 years (*M* = 23.2, *SD* = 2.9). Ninety-seven percent (57/59) of participants were from an urban area, 2% (1/59) from a suburban area, and 2% (1/59) from a rural area. Ninety-five percent (56/59) of participants self-identified as Muslim; 2% (1/59) as Christian-Orthodox; 2% (1/59) as atheist; and 2% (1/59) as deist. Participants’ level of religiosity ranged from 1 to 9 (*M* = 7.46, *SD* = 1.98) on a 10-point Likert scale on which higher scores indicate greater religiosity.

### Measures

The Sleep Paralysis Experiences and Phenomenology Questionnaire (SP-EPQ), devised by the first and last author of this publication (BJ and DH), was administered orally to participants by trained research assistants. The SP-EPQ includes 17 items of which 12 are open- and five closed-ended, and has been used previously in Italy (Jalal et al., 2015, in press). The SP-EPQ is an elaborated version of the Sleep Paralysis Questionnaire (SPQ) that was previously used in Cambodian, Nigerian, Chinese, American, Egyptian, and Danish populations (Hinton et al., 2005a, 2005b; Jalal et al., 2014a, 2014b; Jalal & Hinton, 2013; Ohaeri et al., 2004; Yeung et al., 2005).

The initial item of the SP-EPQ questionnaire asks: “Some people have had the experience upon going to sleep or awakening, when they were unable to move their arms or legs or to speak, even though they wanted to do so. Have you ever had this experience?” To avoid false positives, participants are asked to describe their experience only if they answered affirmatively to this question (Jalal et al., 2014a, 2014b; Jalal & Hinton, 2013). The questionnaire includes questions on the frequency of SP (viz., lifetime, past year, and past month), triggers of the episode, time of occurrence, sleeping position and duration of the event, and related somatic sensations and emotions. It also includes items on causal interpretations, hallucinations and culturally derived meaning, self-treatment and help seeking, and knowledge sources. The SP-EPQ was translated by the second author, a native Turkish speaker (Jalal et al., 2014a, 2014b; Jalal & Hinton, 2013; Ohaeri et al., 2004). To ensure that the Turkish translation of the SP-EPQ was accurate, a back translation of the Turkish SP-EPQ was completed, which was then compared to the original English version; any discrepancies were examined, leading to an alteration of translation.

### Procedure

Participants were Turkish undergraduate college students recruited from İstanbul Şehir University who had previously experienced sleep paralysis. Course credit was offered for participation in the study. Also, a non-random convenient sampling technique, namely, “snowballing” (or chain referral), was used to increase the sample size. We asked participants to refer other individuals to the study (e.g., friends, classmates, and other students) who had previously experienced SP. Participants were informed about the nature of the study and asked to participate. This current investigation was approved by the Institutional Review Board at İstanbul Şehir University (file number: 5/2015), and all participants gave written consent. The SP-EPQ was orally administered to participants by trained research assistants. The SP-EPQ took around 20–25 minutes to administer. All study participants completed the entire interview.

### Data analysis

Data in regard to name associated with SP, causation of SP, sources of knowledge about SP and associated beliefs, hallucinatory experiences, and treating and preventing SP are presented in frequencies and percentages (see too Tables 1–4). The findings are also illustrated in two case studies.

**Table 1.** Sources of knowledge about SP and associated beliefs (N = 59).

	n	%
*Mentioned sources from which they had heard about SP and associated beliefs*	54	92
Family	14	24
Friends	14	24
Various people in their community (excluding family and friends)	4	7
Both family and friends	2	3
Both family and community	1	2
All of the above categories	14	24
Movies	2	3
Newspapers	1	2
Internet	1	2
Religious sources (i.e., a Muslim priest and in religious gatherings)	1	2
*Had talked with someone about their SP experience*	42	7
Had talked with their family	33	56
Had talked with their mother	22	37
Had talked with their father	1	2
Had talked with both their parents	2	3
Had talked with their sister	3	5
Had talked with their cousins	1	2
Did not specify with whom they had talked to from their family	4	7
Had talked exclusively with their friends	2	3
Had talked with both their family and friends	6	10
Had talked with their therapist	1	2
Had talked with both their family and other community members	1	2
*Consulted a traditional or religious healer (a* hodja*)*	4	7

**Table 2:** Hallucinatory experiences during SP (N = 59).

	n	%
*Saw a shadow or being move toward them during their SP*	31	53
“The creature” had ill intentions toward them	24 11	41
Saw a shadow or being sit on their chest	11	19
Saw a dark shadow or a dark human-like figure	24 4	41
Saw a big dark shadow (i.e., an amorphous shadow)	8	14
Saw a dark human figure or a human figure/silhouette	7	12
Saw a person they knew (family, friend, or roommate)	4	7
Saw a big dark shadow turning into a human figure	1	2
Saw a dark shadow with terrifying “nonhuman” vividly blue eyes	1	2
Saw little gypsy children	1	2
Saw a woman with a burnt face and a dark cloud above the bed	1	2
Saw a black object transforming into a face when turning	1	2
Saw a little dog	1	2
Saw a huge round object coming from the walls	1	2
Saw eyes on the wall in an array of bright colors	1	2
Saw a shapeless entity moving toward their head	1	2
Saw a dark (nonhuman) “majestic being”	1	2
Saw a being without feet observing them, leaning on their body, and pressing their chest	1	2
Saw white shapes with faces that flashed in the air and came toward them and a dark-reddish being siting on their chest	1	2
*Sensed an unseen “presence” during SP*	15	25
“This presence” had an evil nature	11	19
*Auditory hallucinations*	14	24
Heard incomprehensible voices	6	10
Heard whispers	3	5
Heard the voice of a person they knew (e.g., mother or brother)	3	5
Heard an unrecognizable human voice or voice of a person they didn’t know	3	5
Heard footsteps	2	3
Heard a breathing voice	1	2
Heard children talking	1	2
*Olfactory hallucinations*	1	2
Smelled burning odors	1	2

**Table 3.** Rate of using treatment techniques for SP among those who used such techniques (N = 31).

Ranked individual treatment methods	*n*	*%*
1. *Dua* (i.e., supplicating to God, which is sometimes done by raising one’s hands in the air with the palms facing upwards)	19	61
2. Physiological preventive measures (e.g., avoiding sleeping after dinner, not eating high calorie foods, taking sleeping pills, leaving one’s bed after SP, drinking water, changing sleeping position, changing one’s pillowcase)	12	39
3. Reciting the Quran (the holy book of Islam)	8	26
4. *Namaz* (i.e., ritualistic prayer, which entails reading verses from the Quran, and involves standing, bowing, sitting, and prostrating)	6	19
5. Psychological prevention techniques (e.g., avoiding thinking about SP, avoiding talking about the *Jinn* and the *Karabasan*, facing one’s fears, having positive thoughts before sleep)	5	16
6. Environmental prevention strategies (e.g., leaving the lights on or leaving the bedroom doors open, sleeping beside one’s parent)	4	6
7. Consulting a *hodja* (an Islamic priest)	2	6
8. Carrying a *musqa* (an amulet with verses from the Quran, usually worn around the neck)	1	3

**Table 4.** Rate of cluster of techniques used to treat SP among those who used such techniques (N = 31).

Cluster of treatment methods	*n*	*%*
1. *Dua* and reciting the Quran (Islam’s holy book)	8	26
2. *Dua* and changing one’s sleep position	4	13
3. *Dua*, *namaz*, and environmental prevention strategies (e.g., leaving the lights on or bedroom doors open and sleeping beside one’s parent)	3	10
4. Physiological preventive measures (e.g., avoiding sleeping after dinner, not eating high calorie foods, taking sleeping pills, leaving one’s bed after SP, drinking water, changing sleeping position, changing one’s pillowcase)	3	10
5. Changed sleeping position and psychological prevention techniques (e.g., avoiding thinking about SP, avoiding talking about the *Jinn* and the *Karabasan*, facing one’s fears, having positive thoughts before sleep)	3	10
6. Psychological prevention strategies (see above for examples)	2	6
7. *Dua* and physiological preventive measures (see above for examples)	2	6
8. *Namaz* only	3	1
9. *Dua* and namaz	3	1
10. Consulting a *hodja* (an Islamic priest)	3	1
11. Consulting a *hodja* and *dua*	3	1
12. *Dua, namaz*, and environmental prevention strategies and carrying a *musqa* (an amulet with verses from the Quran)	3	1

## Results

### Name associated with SP in Turkey

When asked whether they had heard a name for SP, that is, the condition of being unable to move or to speak upon falling asleep or awakening, 95% (56/59) answered affirmatively. Of those, 93% (52/56), that is, 88% (52/59) of all of those surveyed, mentioned *Karabasan*. *Karabasan* refers to a creature in Turkish folklore that is believed to cause paralysis upon falling asleep or awakening, or it can simply refer to the experience of this form of paralysis. Thus, it serves as one way to refer to having SP. In the Turkish language *kara* means “black” and *basan* comes from the word *basmak* which means “to press, to overwhelm” (for details, see discussion section below). Among Turks, it appears there is no clear-cut definition of what the *Karabasan* actually is or what it looks like—other than the fact that it is a supernatural creature. For example, as reported below, a few participants mentioned seeing the *Karabasan* as a person they knew (family or friend) or as an “ugly”-looking human being, indicating that the supernatural being has the ability to manifest in human form.

As elaborated below, among the 93% (56) of participants that referred to SP as *Karabasan*, 79% (44/56) mentioned *Karabasan* only; 9% (5/56) mentioned both *Karabasan* and sleep paralysis; 2% (1/56) mentioned *Karabasan* and nightmare; 2% (1/56) mentioned *Karabasan* and *Jinn*; 2% (1/56) mentioned *Karabasan*, *Jinn*, and described the event “as if there is a monkey on the chest.” Seven percent (4/56) of the participants said there was a name for the condition but did not mention the *Karabasan*, and instead gave one of the following names: sleep paralysis (1/56), apnea (1/56), “being terrorized or frightened” (i.e., by “a *Jinn* or an evil spirit that scares people”) (1/56), and a “*Jinn* attack” (1/56).

### Causation of SP

When asked about causation, 68% (40/59) of participants gave a cause of their SP. Among those giving a cause, 25% (10/40) reported their SP as possibly caused by *Karabasan*, that is, 17% (10/59) of all surveyed. Among the 10 participants that feared the cause to be *Karabasan*, four were certain that their SP was caused by *Karabasan*; five believed that their SP might possibly be caused by either the *Karabasan* or psychological factors, such as stress, fear, anxiety, panic, sadness, guilt, or a depressed mood; and one thought that their SP might be caused by either the *Karabasan* or physiological factors such as fatigue, overeating, thirst, excessive sleeping, and lack of oxygen. Thus, within the participants giving a causal explanation, 15% (6/40) considered their SP to be caused by a combination of supernatural forces (namely, *Karabasan*) and neurophysiological factors; that is, they subscribed to a “dual causal interpretation of SP.”

Seventy-five percent (30/40) of participants that gave a causal explanation for their sleep paralysis reported that their SP was not precipitated by a supernatural cause, but rather by psychological factors, physiological factors, or a combination of these. Of all those giving a causal explanation, 28% (11/40) believed that their SP was caused by stress only; 15% (6/40) that their SP was caused by physiological factors (fatigue, overeating, lack of oxygen, and thirst); 15% (6/40) that their SP was caused by psychological factors (distress, fear, anxiety and feeling depressed, guilt, or sadness); 13% (5/40) that their SP occurred as a result of their sleep position (i.e., sleeping in the supine position); and 5% (2/40) that their SP was caused by a combination of psychological and physiological factors.

### Sources of knowledge about SP and associated beliefs

When queried, overall 92% of participants (54/59) mentioned the sources from which they had heard about SP and associated beliefs (see Table 1). When asked whether they had talked with anyone about their SP experience, 71% (42/59) of participants answered affirmatively. Seven percent (4/59) of all participants mentioned consulting an Islamic priest (a *hodja*^1^) about their SP (see below for a discussion of what these priests suggested as treatments).

### Hallucinatory experiences

Fifty-three percent (31/59) of participants reported seeing a shadow or some being move toward them during their SP. Overall, 77% (24/31) reported that this hallucinated being had ill intentions toward them, and 33% (11/31) mentioned that the visualized being sat on their chest during SP. Twenty-five percent (15/59) of participants reported a “sensed presence” during their SP rather than seeing an actual being, and most of these (73% [11/15]) considered this “presence” to be evil in nature. Twenty-four percent (14/59) of participants reported hearing unusual sounds or voices during their SP. Two percent (1/59) reported smelling a burning odor during the SP episode. (For details see Table 2.)

### Treating and preventing sleep paralysis

Out of all participants, 53% (31/59) took measures to prevent SP, and of these, 71% (22/31) applied various religious and cultural prevention techniques that sometimes combined with “non-supernatural” means, and 29% (9/31) resorted to only “non-supernatural” preventive remedies. The frequency of the techniques used by the participants is listed in Table 3. As can be seen, *dua* (supplicating to God, described below) was the most frequent, used by 61% of those who tried to prevent SP, followed by physiological means (e.g., not sleeping after dinner), used by 39%, followed by other techniques, many religious in nature: reciting the Quran, *namaz*,^2^ consulting a *hodja* (an Islamic priest), or carrying a *musqa*. We also determined the clusters of interventions participants employed and ranked those clusters in terms of frequency, which is shown in Table 4.

We specifically asked whether the participants visited either a traditional healer or religious healer, and we found that 7% (4/59) of participants had consulted a *hodja*, who also serve as traditional or religious healers in Turkey. These *hodjas* had suggested the following “prevention techniques”: *(1) Dua* (supplicating to God) (50% [2/4]). In the Islamic faith, *dua* refers to supplicating to God for one’s physical and spiritual needs. *Dua* sometimes entails raising one’s hands in the air, with the palms turning upwards, while one is supplicating to God. (2) Carrying a *musqa* (i.e., a good luck talisman inscribed with Quranic verses or Islamic prayers often carried around the neck as a necklace) (50% [2/4]). (3) Reciting the *azan*^3^ (the Islamic call for prayer) (25% [1/4]). (4) Reciting *Esmaul Husna* (*Allah*’s Holy names; for details on Allah’s names and their therapeutic relevance see, Jalal et al., 2017) (25% [1/4]).

### Case studies of SP

#### Case 1: Sleep paralysis as “spiritual punishment”

Begüm is 21 years old and has experienced SP twice. She interprets her SP as “*Karabasan*.” On one occasion, she was sleeping on the couch and suddenly realized she was paralyzed. She noticed the TV was on, but all she could hear were incomprehensible voices. Her family was next to her—and she desperately wanted to scream out, but to no avail—she couldn’t produce the slightest sound! Indeed, Begüm felt she was floating in a twilight zone between fantasy and reality. At this point, she started to fear that she might be dying.

Begüm believes that SP happens as a result of her neglecting her religious duties, such as *namaz* (i.e., ritualistic prayer) and *dua* (supplication). As such, she regards SP as a form of “spiritual punishment” for not abiding by her religious faith. During another SP episode, she again thought she was dying, and kept ruminating over the fact that she had neglected her *namaz*, which generated more guilt, in turn exacerbating panic and worry during the event.

She has shared these frightening experiences with several people, including her mother and her therapist. While her therapist thinks that a combination of Begüm’s guilt and anxiety triggers her SP, her mother is convinced that SP occurs due to her neglecting the *namaz*. Indeed, for Begüm, SP was “spiritual punishment” and gave rise to great guilt.

#### Case 2: A horrifying Karabasan

Zeynep, 23 years of age, has experienced SP five times throughout her life. She first heard about the “*Karabasan”* from her grandmother when she was only seven years old. Zeynep’s SP episodes tend to occur when she is anxious, upset, or stressed. She experiences various hallucinations during SP, but her last episode stood out as particularly petrifying: Zeynep woke up in a state of paralysis, and had the catastrophic thought that her paralysis might be permanent. Then, suddenly, out of nowhere, a dreadful-looking woman appeared before her—whom she immediately recognized to be the *Karabasan*. The *Karabasan* had a foul burning odor and a burned face. In a creepy gabbling voice, the creature started to call out to Zeynep. Utterly horrified, Zeynep feared the *Karabasan* might touch her; she was convinced that merely being touched by this horrendous creature would in fact transform her into a woman with a burned face.

Zeynep mentioned this dreadful experience to her parents, who advised her to consult both a psychiatrist and a *hodja* (an Islamic priest). She took their advice and saw a *hodja*, who told her that she had indeed been assaulted by a *Jinn*. The *hodja* went on to write a *musqa* for Zeynep (a talisman of sorts containing Quranic verses and Islamic prayers) that she could carry around her neck at all times as protection against *Jinn* attacks. In addition to wearing this talisman, Zeynep engaged in prayer and Quranic recitation before going to bed as a way of preventing SP attacks (and occasionally took sleeping pills to help her sleep better).

## Discussion

In the current study, we found that when Turkish college students were asked whether they had heard a name for SP, the vast majority (88%) mentioned attack by the *Karabasan*. The fact that the vast majority of participants mentioned the *Karabasan* as the name they knew for SP—a highly specific cultural name—suggests that the cognitive category for SP is very much embedded in present-day Turkish culture; that is, the phenomenon is well known and has cultural salience. When asked about the sources of their knowledge about SP and associated beliefs, 54 of 59 participants (92%) identified a source, with participants often identifying their families (26% or 14/54) or friends (26% or 14/54) as the primary source.

Though the vast majority (88%) of the college students used the term *Karabasan* as a way to describe SP, only 17% believed that their SP might have been caused by this supernatural creature. However, it is possible that many more believed that the *Karabasan* was the cause of their SP, but were perhaps too embarrassed to mention these fears. The fact that such a high percentage of those with SP hallucinated a “malicious visitor” speaks to this fact: 41% saw a shadow or being move toward them that was thought to have a malicious intent. It appears that supernatural beliefs about SP are widespread in Turkey. In some other modernized countries, this has also been found; for example, 38 percent of Italians from the general population—a highly modern country—believe that their SP might have been caused by the *Pandafeche*, which is described there as a witch or supernatural cat-like creature (Jalal et al., 2015).

Whereas 27% of Turkish college students thought their SP might be caused by a specific supernatural creature, an even greater number of Turks from the general population would perhaps give a supernatural explanation of SP. Highly educated Turkish students who are scientifically literate are more likely to be influenced by Western socio-cultural frameworks. There might also be an expectation bias in which university students are expected to adhere to scientific principles, making them less likely to admit to supernatural beliefs in general (see also Jalal & Hinton, 2015). One study found that Egyptian college students are much less likely to admit to believing in supernatural causes of their SP compared to Egyptians from the general population (11% vs. 71%) (Jalal et al., 2014a). Similar to these Egyptian college students, participants in the current study (students at Şehir University in İstanbul) are instructed in the English language; and as we have mentioned elsewhere, access to the English language in particular might provide an avenue for exposure to Western culture and science, for instance via the internet (Jalal et al., 2014a). On the other hand, our participants were predominantly Muslim (95% as reported above), many of whom were religious (overall participants scored a mean of 7.47 out of 10 in terms of religiosity levels), so this might have influenced reporting as well.

It is conceivable that SP rates might have been higher if the study had been conducted in another city or a more rural area. Turks from other cities and especially from rural areas of Turkey might be more likely than our participants (Turkish students from İstanbul) to ascribe to cultural beliefs about SP. İstanbul is a cosmopolitan city that geographically and culturally functions as a bridge between the continents of Europe and Asia. The city has a plethora of cultures, ethnicities, and languages, and is regularly frequented by a large number of tourists from around the globe. Indeed, one finds both Western and Eastern elements in the local culture.

In the present study, participants also reported a dual causal view of SP. This is the belief that SP is caused both by supernatural events as well as naturalistic factors (e.g., brain physiology). Such dual causal views about SP have also been reported in Egypt, Denmark, Italy, and the United States (Hufford, 2005; Jalal et al., 2014a, 2015). Such a dual view often comes about as SP experiencers attempt to reconcile scientific and spiritual explanations of SP; for instance, by incorporating scientific explanations into their already established supernatural beliefs about the experience. Indeed, given the uncanny nature of SP, it is unsurprising that experiencers have a hard time shaking off their supernatural beliefs about the experience.

The fact that there is no clear definition of what the *Karabasan* is or what it looks like is interesting. This is similar to the traditional Islamic understanding of the *Jinn*: there is no clear definition of what the *Jinn* looks like in its original form, and it likewise can adopt the form of a human (usually an unattractive one). Indeed, given that Turkey is a predominantly Islamic country, it is unsurprising that beliefs about the *Karabasan* and the *Jinn* overlap. This is consistent with Turkish folk tradition, where the *Karabasan* is sometimes believed to be a specific type of *Jinn* (see Amer & Jalal, 2011; Jalal et al., 2014a), though others believe the *Karabasan* to be a separate supernatural creature altogether. By way of contrast, in Italy, which is not a Muslim majority country, the creature that in the folklore is thought to sometimes cause SP, namely the *Pandafeche*, has a specific physical appearance—and usually presents itself as either a witch or a cat-like creature.

In the present study, participants almost always referred to SP as *Karabasan*, with a few people also using the term *Jinn*. However, there are other names and cultural beliefs associated with SP reported in Turkish folk-literature. One example is the *Ağırlık* (literally translated as “heaviness”). According to the folk-literature, this is another name for the *Karabasan* creature, which is known for throwing itself onto people while they are sleeping and pressing down on them with full force to prevent them from awakening. It is thought to be an evil creature that can strangle people to death. Yet another cultural creature that might cause SP according to Turkish folk tradition is the *Kamos*. *Kamos*, which is believed to resemble the *Karabasan*, sits on sleeping people, causing them to die or to become possessed. It is sometimes described as a giant or a dwarf. This evil creature can be seen taking the shape of a black cat. *Kamos* always wears a *börk* (a traditional Turkish hat which is made of leather) and it is believed that if a person is able to catch the *Kamos*’s *börk*, then the *börk* turns into gold (Duvarcı, 2005). From Turkish folk-literature, it is evident that there are numerous elaborate cultural traditions about SP in Turkey. Our data suggest that the *Karabasan* expression, and to some degree associated cultural beliefs, are an experientially salient part of modern-day Turkey, whereas other names for SP seem to be less prevalent. More research is needed to clarify the prominence of local frames for understanding SP. It may be that these other terms, like the terms “incubus” and “succubus” for SP in the West, may now be rarely used, with the term “*Karabasan*” becoming the salient frame.

The hallucinatory experiences during SP that are reported in this study are consistent with reports from other cultures. For instance, more than half of the participants (53%; 31/59) reported seeing a shadow or being move toward them during their SP; in Italy, 38% of experiencers saw such a shadow. The highest reported rates of the hallucination are among traumatized Cambodian refugees, with a rate of 90% among those with SP (Hinton et al., 2005b). This particular visual hallucination is intriguing, and leads to much local cultural interpretation. The frequency with which this visual hallucination occurs around the world speaks to its robust neuro-phenomenology, driven by precise neurological triggers (Cheyne et al., 1999a, 1999b; Cheyne & Girard, 2009; Jalal & Ramachandran, 2014, 2017).

We also found that almost one in four participants (24%) reported hearing unusual sounds or voices during their SP and that more than one in five participants (22%) hallucinated a shadow or being sitting on their chest (19% or 11/59) during the event. Moreover, a quarter of the participants (25%) had a “sensed presence” hallucination. These hallucinations are also commonly reported by SP experiencers worldwide (Jalal & Ramachandran, 2014; Jalal et al., 2014a), and might similarly arise from deafferentation of sensory signals that, combined with threat hypervigilance and hippocampal activation (e.g., memories of suffocation sensations), create the illusion of an exogenous threat. In line with other cultural contexts, almost three out of four of those who felt a “sensed presence” in this study reported that this “presence” was evil in nature (Jalal & Ramachandran, 2014; Jalal et al., 2014a).

In this study, 7% of participants (4/59) consulted an Islamic priest (a *hodja*) about their SP. By way of comparison, 26% (11/42) from the general population of Egypt who attributed SP to the *Jinn* consulted a religious priest (a *Sheikh*) about their experience (Jalal et al., 2014a). While the Egyptian *Sheikh* mainly recommended Quranic recitation (90% or 10/11) and performing prayers five times a day (55% or 6/11) as preventative remedies against SP, the local *hodja* in Turkey recommended a wider range of preventive strategies including reciting the Quran and the *azan* (i.e., the call for prayer); evoking *Esmaul Husna* (Allah’s holy names); performing the five daily prayers; and carrying a *musqa* (a talisman). On the other hand, Italians (Jalal et al., 2015) and Danes from the general population (Jalal et al., 2014a) did not consult religious figures or spiritual healers about their SP (except one Italian individual who consulted a Catholic monk). Taken together, these findings suggest that approaching religious or spiritual healers about SP might be a relatively common phenomenon in Islamic cultures, whereas it might be rarer in Western or European contexts. The present results also shed light on which Islamic healing techniques might be most common and also how these differ from one Islamic culture to another (i.e., Egypt vs. Turkey), with Turkish *hodjas* ostensibly being more pluralistic in their approach to spiritual healing.

We found that 37% of participants (22/59) applied supernatural and religious prevention techniques—occasionally combined with “non-supernatural” means (e.g., sleeping pills and avoiding high calorie food)—to protect against SP. The most popular spiritual healing technique was engaging in *dua* (supplicating), which was practiced by 61% of students trying to prevent episodes (see Table 3). *Dua* was often combined with reciting the Quran for well-being (see Table 4). The fact that more people resorted to supernatural remedies than individuals actually admitting to supernatural explanations of SP (25%) raises important questions. It strongly indicates that participants were perhaps not willing to admit to believing in supernatural causal explanations (we have provided some reasons above for why this might be the case). Moreover, Islamic healing, known as *ruqyah* (derived from the Quran and the teachings of Prophet Muhammad), is traditionally used as treatment for all types of ailments and disease, including those thought to be spiritual (e.g., demonic possession by *Jinn* or the “evil eye”) and naturalistic in nature (say, cancer or a stomach infection). More generally, it is noteworthy that participants often would combine supernatural treatments with traditional medical approaches. This indicates that the participants viewed the two forms of treatment as complementary rather than as antithetical to one another. Moreover, more than half of the participants (53% or 31/59) took some type of measure—whether supernatural, naturalistic, or both—to prevent future episodes of SP. Similar findings have been reported in Italy, where 35% of individuals who experienced SP took some kind of measure to prevent SP from reoccurring. That such a large a percentage of SP experiencers take steps to prevent future episodes of SP highlights the distressing nature of SP and the need for evidence-based clinical treatments for it (e.g., Jalal, 2016; Sharpless & Doghramji, 2015).

The current study has broader culture-sensitive treatment implications. Given the number of Turkish SP experiencers in the present study who conceptualized SP in terms of a *Karabasan* attack and/or applied supernatural and religious prevention techniques when dealing with SP, it is crucial that evidence-based mental health services in Turkey (which are often modelled after Western treatment approaches, and thus tend to be secular per se; on community mental health services in Turkey, see Gökalp & Aküzüm, 2007; see also Mocan-Aydin, 2000) take such multi-layered cultural beliefs into account in the treatment of sleep and nightmare related issues, including repetitive and distressing sleep paralysis. Moreover, there are currently a great number of Turks living outside Turkey, especially in European countries, where many originally arrived as guest-workers (i.e., economic migrants) in the 1970s and since have brought their families with them; for instance, there are over 2.1 million Turkish immigrants living in Germany alone (Ehrkamp & Leitner, 2003). Psychiatrists and psychologists in these countries should likewise keep such culturally sensitive issues in mind when working with Turkish patients who suffer from fearful and chronic SP (i.e., raise their standards of cultural competence, such that these pervasive cultural beliefs are not ignored and can be appropriately addressed).

The study has limitations. The sample size was small, and undoubtedly a larger sample size would have helped shed better light on the nature of the *Karabasan* and associated beliefs. Additionally, the fact that this study was conducted among students in İstanbul, many of whom were religious, might give us a skewed impression of the actual presence of cultural beliefs about SP and the *Karabasan* in Turkey more broadly.

## Conclusion

In summary, we found that 88% of participants mentioned the supernatural creature the *Karabasan* when asked whether they knew a name for SP. Moreover, 17% of the participants believed that their SP might have been caused by this supernatural creature. We found that 37% of our participants, college students in Turkey (a highly secular country), apply various supernatural and religious approaches to prevent future SP attacks, including *dua* (supplicating to God), reciting the Quran (the holy book of Islam), and wearing a *musqa* (a type of talisman inscribed with Quranic verses). In short, we found that the *Karabasan* constitutes a culturally specific interpretation of the phenomenology of SP in Turkey, and provides a term to describe SP, leading to cultural salience of the experience. Future research should disentangle precisely how the *Karabasan* explanation of SP found in Turkey differs from the *Jinn* attack explanation found in other Islamic cultures, such as Egypt.

## Notes

1. The term “*hodja*” in Turkey refers to Islamic priests that lead the congregational prayer in the mosque and have a leadership position in the Muslim faith-community (e.g., are knowledgeable in Islamic scriptures). In Islamic cultures, Muslim priests such as the *hodja* can sometimes serve the role of a religious healer.

2. According to mainstream Islamic beliefs, Muslims are to perform ritualistic prayer five times a day, which involves standing, bowing, and prostrating.

3. The *azan*, the Islamic call to prayer, is called from the minaret of a mosque five times a day to announce that it is time to perform the obligatory Islamic prayer, known as the *salah* or *namaz* in Turkish.
